# Cone Beam CT Features and Oral Radiologist’s Decision-making of Arrested Pneumatization of the Sphenoid Sinus

**DOI:** 10.2174/1573405619666221130115929

**Published:** 2023-05-17

**Authors:** Noura Alsufyani, Nouf Alsuayri, Raghad Alrasheed

**Affiliations:** 1 Oral & Maxillofacial Radiology, Oral Medicine and Diagnostic Sciences Department, College of Dentistry, King Saud University, Riyadh, Kingdom of Saudi Arabia;; 2 School of Dentistry, Department of Medicine and Dentistry, University of Alberta, Canada;; 3 Dental University Hospital, King Saud University, Riyadh, Kingdom of Saudi Arabia

**Keywords:** Sphenoid sinus, development, cone beam CT, skull base, diagnostic imaging, APS

## Abstract

***Objectives*:** To assess the demographic and radiographic features of arrested pneumatization of the sphenoid sinus (APS) and their influence on the confidence of oral and maxillofacial radiologists (OMFRs) in diagnosing APS.

***Methods*:** Reports of cone beam computed tomography (CBCT) APS were retrieved, and the demographic and radiographic features were retrospectively analyzed. Five OMFRs assessed the CBCT images and their confidence in diagnosing APS. The OMFRs’ experience (years), expertise (skull-base CBCT cases/month) and diagnostic confidence level were analyzed for agreement and associations with demographic or radiographic features.

***Results*:** Of 29 APS cases, 17 (58.6%) were females, and the mean age was 29.9±19 years. Twenty cases (69.0%) presented unilaterally, and 27 (93.1%) involved the sphenoid body. The most common accessory site was the pterygoid process (19, 65.5%). The vidian canal and foramen rotundum were involved in 27 (93.1%) and 17 (58.6%) cases, respectively. Most cases (28, 96.6%) were well-defined, corticated, and showed mixed attenuation. APS diagnostic confidence was higher among the expert OMFRs (72.4%-82.8% *vs*. 58.6%-62.1%).

***Conclusion*:** Radiographic features differentiating APS from skull-base tumors were shown on CBCT. The confidence of OMFRs with similar experience in years depended on their frequency of examining CBCT cases involving the skull base.

## 1 INTRODUCTION

The sphenoid sinus is one of four paranasal sinuses. It is bordered by important anatomical structures: ethmoidal air cells anteriorly, the basiocciput (clivus) posteriorly, the cavernous sinuses laterally, the pituitary fossa superiorly, and the nasal cavity inferiorly. Important neurovascular structures lie near the sphenoid sinus, such as the intracranial internal carotid artery, optic nerve, and cranial nerves III, IV, V2, and VI.

Pneumatization is a lengthy and complicated part of the developmental process of paranasal sinuses that starts in utero at 3 or 4 months and continues throughout adulthood [1]. At birth, the sphenoid sinus is filled with red bone marrow (hematopoietic tissue), then begins conversion to yellow bone marrow (adipose tissue) at the age of 4 months [2]. The cause of conversion is unclear, but some studies have proposed temperature and vasculature changes, oxygen tension, and chronic inflammation (cystic fibrosis) [1, 3]. By the second year, conversion is complete, and the respiratory mucosa is formed [1, 3, 4]. Later, aeration completely pneumatizes the sinus by the age of 12-14 years [1]. When pneumatization is interrupted at any point, adipose tissue will persist through adulthood, resulting in arrested pneumatization of the sphenoid bone (APS) [1].

The reason arrested pneumatization occurs more frequently in the sphenoid sinus than in other paranasal sinuses is undetermined but might be related to the high variation in the extent of pneumatization [2]. The prevalence of APS is 0.7%-4.8% in normal subjects [5-7] and 4.6%-9.6% in subjects with hematological conditions (*e.g*., sickle-cell anemia, thalassemia, and leukemia) [3, 7]. The elevated serum erythropoietin levels in hemoglobinopathies can cause the yellow marrow to reconvert to red marrow and interfere with the pneumatization process [7].

APS is considered underreported and mistakenly confused with another skull-base pathosis, and medical practitioners and surgeons are unfamiliar with this entity [3, 7, 8]. The radiographic features can closely mimic other benign or aggressive skull-base lesions, such as intraosseous lipoma, intraosseous hemangioma, fibrous dysplasia, chordoma, arachnoid granulation, metastatic lesion, meningioma, Paget’s disease, and chronic inflammation, such as osteomyelitis [1, 4].

There are several studies on the pattern of normal pneumatization and size of the sphenoid sinus; however, APS is not thoroughly investigated in the literature [9-12]. Sixteen published studies have described the computed tomography (CT), magnetic resonance imaging (MRI), or cone beam CT (CBCT) features of APS [1-8, 13-20]. All except one [2] were case reports, small case series, or focused on populations with hematopoietic diseases. Only four case reports were based on CBCT [1, 4, 17]. All of these reports stated that it is important to distinguish APS from any other lesion and avoid unnecessary investigative surgeries or biopsies [1, 19]. However, the awareness and confidence of radiologists in diagnosing APS are unclear and never tested. When the radiologist is confident about the interpretation, definitive clinical decisions can be made more easily. The fear of making an error is problematic for radiologists and could contribute to unnecessary testing or intervention [21-23].

Sinus pneumatization and variations can change surgical planning [10]. However, arrested pneumatization adds a diagnostic dilemma to the radiologist, and the literature addressing this challenge is lacking.

The aims of this study were:

• To assess the demographic and radiographic features of APS to provide a wider understanding of its radiographic presentation on CBCT.

• Test the decision-making of oral and maxillofacial radiologists (OMFRs) in diagnosing APS.

• Identify the demographic and radiographic features that may influence the diagnostic confidence of OMFRs.

## 2 MATERIALS AND METHODS

### 2.1 CBCT Reports and Images

This retrospective analysis screened radiographic reports, and CBCT images of dental patients scanned for maxillofacial purposes at two imaging centers in Edmonton, Alberta, Canada. Approval was obtained from the Health Research Ethics Board (ID # Pro00102037) at the University of Alberta and complied with the Helsinki Declaration, and each subject in the project signed a detailed informed consent form. The authors verify compliance with the Health Insurance Portability and Accountability Act of 1996 (HIPAA). The radiographic reports in the database were electronically searched for the words “arrested, ” “pneumatization, ” “sphenoid, ” “sphenoid sinus, ” and “skull base.” The resulting reports and corresponding CBCT volumes were screened by the principal investigator (PI) (OMFR: 11 years of experience) for inclusion in APS. The included APS cases were reviewed by two examiners (NS and RF: dental interns) to extract the radiographic features and then confirmed by the PI. The CBCT images were acquired on an iCAT^®^ Next Generation CBCT system (Imaging Sciences International Inc., Hatfield, PA, US) with a medium-to-large field of view, 120 kVp, 20 or 35 mAs, and 0.3-mm or 0.25-mm voxel size depending on the original purpose of imaging.

### 2.2 Clinical and Radiographic Features

Demographic data included age and sex. The radiographic features were location (unilateral *vs*. bilateral, sphenoid body, greater or lesser wings, pterygoid processes, and involvement of the vidian canal, foramen rotundum, foramen ovale, carotid canal, or foramen spinosum), periphery (definition and cortication), the internal structure (high, low, or mixed attenuation), and effects on surrounding structures (expansion of cortical boundaries, narrowing, or displacement of foramina).

### 2.3 OMFR Confidence

To test the confidence level among the OMFRs for APS diagnosis, five OMFRs examined the CBCT images. The OMFRs were asked to record their diagnosis of ASP. If the OMFR diagnosed the cases as APS based on the CBCT appearance, the outcome was recorded as “confident.” If the OMFR could not diagnose APS and required further imaging, the outcome was recorded as “not confident.”

In the context of this study, confident OMFRs were grouped as “experts, ” and OMFRs with low confidence were grouped as “non-experts.”

To understand the background of each OMFR, their experience (measured by years spent in practice) and expertise (measured by the number of CBCT cases of skull base they reviewed per month) were collected.

### 2.4 Statistical Analysis

All statistical tests were performed in IBM SPSS Statistics for Windows, version 28 (IBM Corp., Armonk, N.Y., USA). Descriptive data analysis was done by frequency of qualitative variables and the mean and SD for quantitative data. The level of OMFR confidence in diagnosis was analyzed by assessing the Kappa inter-rater agreement. The McNemar test was used to assess the difference in OMFR confidence based on experience or expertise. Logistic regression analysis was performed to determine the associations between the absolute agreement of the OMFRs’ confidence with the demographic data and radiographic features of APS.

## 3 RESULTS

There were 29 reports with APS cases. The original reasons for imaging were temporomandibular joint disorders (n = 12), pre-orthognathic surgery (n = 9), and maxillary implant (n = 8). The mean age was 29.9 ± 19 years (range: 7-68 years), and there were 17 (58.6%) females.

**Fig. (1):**
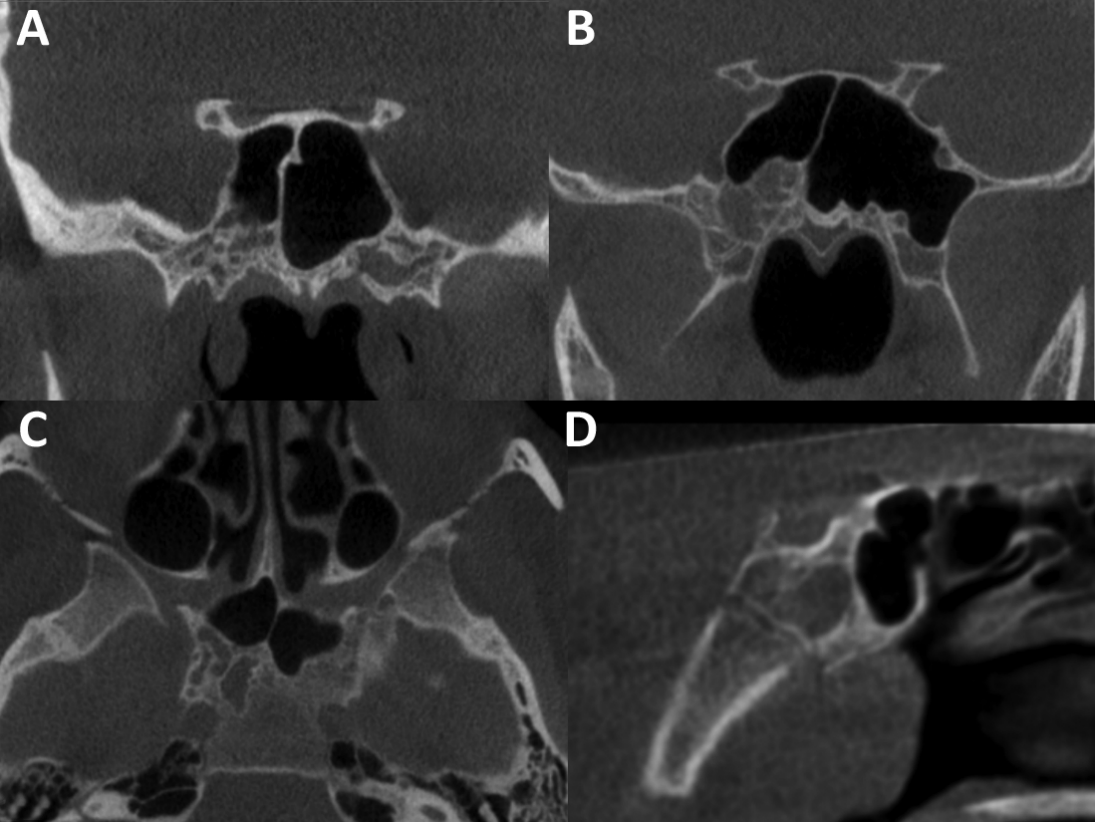
Examples of arrested pneumatization of the sphenoid (APS). Well-defined, sclerotic border, variable distributions of low-attenuation areas, and curvilinear calcifications in coronal CBCT images with (**A**) bilateral APS in the body and greater wings of the sphenoid, (female, 40 years), (**B**) right APS involving the pterygoid process (female, 13 years). (**C**) Axial section of APS in the right body of the sphenoid (female, 32 years). (**D**) Sagittal image showing unusual lack of curvilinear calcification and presence of spheno-occipital synchondrosis (male, 7 years).

The radiographic features of APS are shown in Table **1**. APS mostly presented unilaterally (n = 20, 69.0%) and involving the sphenoid body (n = 27, 93.1%) (Fig. **1A-D**).

**Fig. (2):**
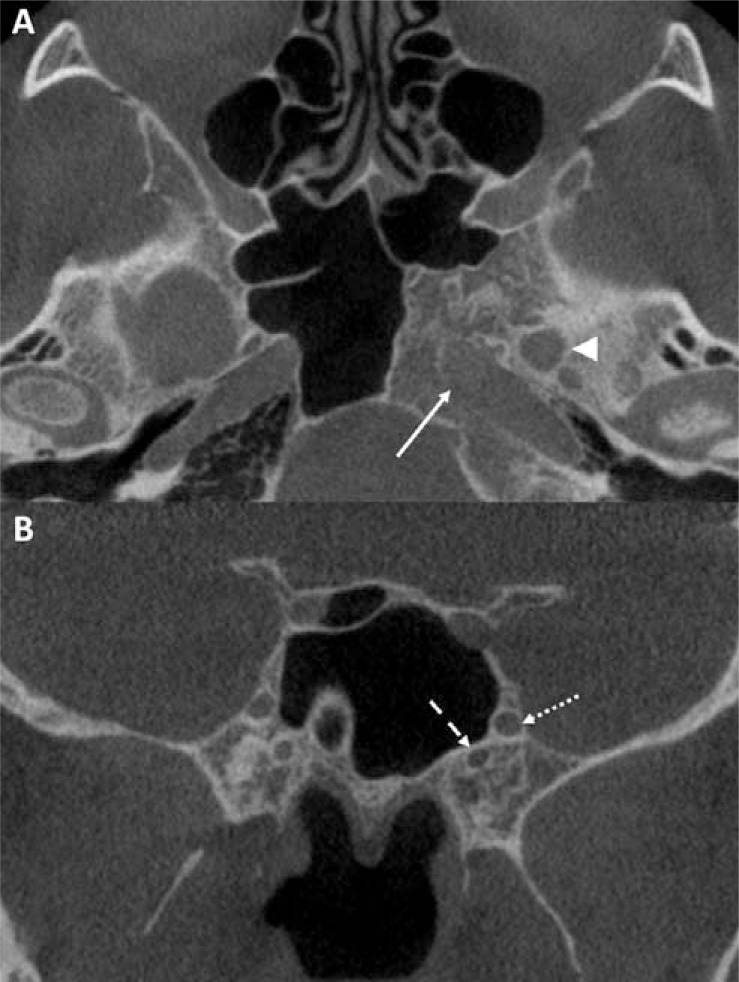
Examples of anatomical structures involved in arrested pneumatization of the sphenoid (APS). No effects on the surrounding structures were noted in (**A**) axial CBCT image with APS extension to the left foramen ovale (*arrowhead*) and the carotid canal (*solid arrow*) (male, 45 years), and (**B**) coronal CBCT image of left APS extension to the foramen rotundum (*dotted arrow*) and vidian canal (*dashed arrow*) (male, 48 years), note incidental dense bone island in the right sphenoid.

The most common accessory site was the pterygoid processes (n = 19, 65.5%). The most common structure involved was the vidian canal (n = 27, 93.1%), followed by the foramen rotundum (n = 17, 58.6) (Fig. **2A, B**).

**Fig. (3):**
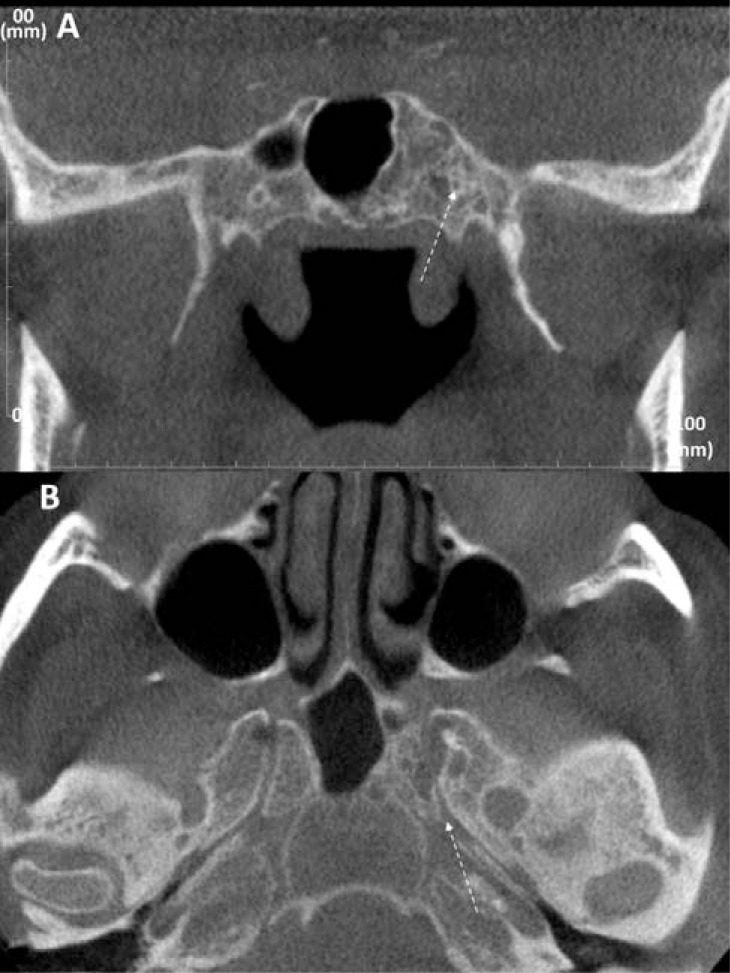
Example of arrested pneumatization of the sphenoid (APS) affecting the vidian canal. The left vidian canal (*dashed arrow*) is smaller in (**A**) coronal CBCT image and irregular in (**B**) axial CBCT image than the right canal, but the cortical outline of the canal is largely maintained (male, 61 years).

The APS in all cases except for one was well defined, corticated, and showed mixed attenuation (Fig. **1D**). Only one case showed mild narrowing of the vidian canal (Fig. **3A, B**).

All five OMFRs had >10 years of experience. OMFR1 and OMFR2 reported viewing >20 CBCT cases of skull bases/month, OMFR3 reported >30 cases/month, and OMFR4 and OMFR5 reported <10 cases/month. The results of inter-rater agreement and confidence in OMFR diagnosis are summarized in Table **2**. The confidence in diagnosis was higher for OMFR1, OMFR2, and OMFR3, who frequently viewed images of the skull base (*i.e*., experts) (72.4%-82.8%) than for OMFR4 and OMFR5 (*i.e*., non-experts (58.6%-62.1%). Inter-rater agreement was substantial within the separate OMFR subgroups; expert *vs*. not-expert (Kappa range: 0.70-0.79). The agreement dropped to *none* when considering an expert examiner with a non-expert examiner (Table **2**). The absolute agreement of APS diagnosis (*i.e*., all examiners agreed they were confident) in the “expert” examiner group was n = 23 (79.3%) *vs*. n = 15 (51.7%) in the non-frequent examiner group (*p* = 0.05).

Logistic regression analysis results of ASP diagnosis based on the absolute agreement of the expert OMFRs are presented in Table **3**. An expert OMFR was not confident in diagnosing APS when the case was a child, involved skull foramina, lacked extension to accessory sphenoid sites, or if it was unilateral (*p* > 0.05).

## 4 DISCUSSION

The median age of the subjects with APS was 23 years, which is younger than what was reported previously (30-44 years) [2, 7] because of the higher percentage of pediatric subjects (<18 years old) in our study (n = 14, 48.3%). APS has been reported in the young population (≥7 years) [3, 5, 15, 18]. This study showed a slight female predilection of 58.6%, whereas Welker *et al*. [2] showed a higher frequency in males (56.6%). Considering all of the APS case reports [1, 3-8, 13-20], the female: male ratio was 5:3.

The sphenoid body is commonly affected (27, 93.1%), similar to the findings of Welker *et al*. [2] (80%) and case reports specifying the APS location (20/25, 80%) [1, 4, 5, 8, 13-15, 17, 19]. The most common accessory site was the pterygoid process (65.5%). Involvement of the pterygoid process and greater wings of the sphenoid in APS were common [1, 2, 4, 5, 7, 8, 15, 16, 18, 19]. Uncommon accessory sites were the clivus, anterior clinoid process, and petrous-temporal bone [2, 13, 14, 16]. The location of arrested pneumatization seems to reflect the location of normal pneumatization. Studies showed that the most common pattern of sphenoid pneumatization is in the body of the sphenoid (27.7-47%), followed by the pterygoid process (17.7-33.1%) [9-12]. Normal pneumatization of the sphenoid sinus usually extends to or slightly beyond the sella; as such, arrested pneumatization is not likely found in uncommon accessory sites [9-12].

APS presented mostly unilaterally (69%), which agrees with the literature. Bilateral cases present at the midline and extend bilaterally in larger cases [7].

The foramen rotundum is located in the middle cranial fossa at the base of the greater wing of the sphenoid and runs infero-laterally toward the pterygopalatine fossa. The vidian canal (pterygoid canal) is located in the pterygoid process of the sphenoid bone and is infero-medial to the foramen rotundum. APS extended to both anatomical structures in 93.1% (vidian canal) and 58.6% (foramen rotundum) of the cases. Only one case extended to the foramen ovale and foramen spinosum. Few previous reports have commented on the extension to surrounding foramina, and their results similarly extended to the vidian canal [1, 2, 8, 13, 18] or foramen rotundum [1, 2]. The extent of APS to the foramen rotundum and vidian canal seems to follow normal pneumatization of the sphenoid. A statistically significant correlation was found between pterygoid pneumatization, vidian canal and foramen rotundum protrusion [11].

Welker *et al*. described characteristic CT features of APS: well-defined, sclerotic border, low attenuation with curvilinear radiopacities, and no effects on surrounding structures [2]. The internal structure consists of fat patches and soft-tissue densities. This study revealed that 96.6% of the cases had the features described above except for one case with an ill-defined border and narrowing of the vidian canal. Due to the low soft-tissue contrast of CBCT, fat densities are not discernable. The histological features explained the radiographic features and showed fatty or fibro-fatty tissue with bone that lacked osteoclastic activity [8, 14, 19]. Most APS cases in the literature conform with Welker *et al*.’s [2] features, but others do not because they lack curvilinear radiopacities [1, 2, 18], have eroding cortical boundaries [1, 14], or exhibit slight expansion [18]. The common MRI features of APS are high signal intensity in T1 (confirming fat content) and heterogeneous in T2, with no enhancement in fat-saturated post-gadolinium T1 [1-3, 5, 8, 13, 15-20]. However, some MRI studies of APS report low signals in T1 [2, 17] or enhancement in fat-saturated post-gadolinium T1 [2, 19]. Based on the CBCT features of APS (similar to those of Welker *et al*. [2] except for fat content), small intraosseous lipoma, intraosseous hemangioma, or fibrous dysplasia would be considered in the differential diagnosis. Further assessment with multidetector (MD) CT or T1-weighted MRI would confirm fat content; thus, the remaining differential diagnosis is an intraosseous lipoma, for which growth monitoring is recommended [24]. Suppose one of the radiographic features is not present, especially its effects on surrounding structures. In that case, other entities, such as chordomas, arachnoid granulations, metastatic lesions, meningioma, Paget’s disease, and chronic inflammation, such as osteomyelitis, can be considered. Requesting MDCT and/or MRI is justifiable, and biopsy is rarely needed. Only three APS cases in the literature were biopsied due to severe headaches or cancer of the lymphoid tissue with ill-defined borders [8, 14, 19]. Two case reports described follow-up, with no change in 7 years without intervention [15], and an increase in aeration 4 years after biopsy [19].

All participating OMFRs had similar experiences measured by years in practice. However, based on the number of CBCT cases of skull base per month, their expertise was different. OMFRs who viewed <10 cases/month were not confident that the entity was APS and preferred to request further imaging in 37.9%-41.4% of the presented cases, whereas expert OMFRs, who are exposed to more skull-base imaging, recommended additional imaging to decide 17.2%-27.6% of the cases. This difference, however, was not significant (*p* = 0.05).

Radiologists’ errors are generally perceptive or cognitive. Perceptual error is more common (60-80%) and occurs when the radiologist fails to identify the abnormality, *i.e*., scanning error. Cognitive errors are less common (20-40%) and occur when the radiologist identifies the abnormality in the scan but fails to correctly understand or report its significance, *i.e*., misinterpretation [25]. The stress of falling into cognitive error impels underconfident radiologists to request additional studies, which increases costs [25].

The logistic regression analysis (Table **3**) examined four variables that could affect the expert OMFR’s confidence in APS cases. The lower confidence level in diagnosing APS when the case was a child is likely due to the thin and loose periosteal layer (lacks cortical definition), spheno-occipital synchondrosis, and lower scan resolution (lower-dose protocols). The involvement of skull foramina and the unilateral location of APS renders the diagnosis challenging and gives a false impression that the lesion is more likely to be a tumor pathology rather than an anatomical variant. The confinement to the sphenoid body could reflect a small lesion size with few radiographic features to interpret *vs*. extension to accessory sites. The odds ratios (OR) in the regression model had large 95% confidence intervals; the OR did not show statistically significant differences [25, 26]. Cognitive errors in underconfident radiologists can be improved by education, simulation training, and access to follow-up results to verify final diagnoses [23, 25, 26].

The main shortcoming of this study was the sample size which limited the statistical tests. Another limitation is the lack of MDCT or MRI to corroborate the fat content. However, fat content is seldom missing, and the cases included had the other important features of APS. The current study did not test the perceptive errors of the OMFRs because the purpose was to identify the demographic and radiographic features that could decrease the OMFRs’ confidence in diagnosing APS on CBCT images.

Future studies should aim to test the perceptive and cognitive errors in diagnosing ASP by including entities similar to ASP and increasing the sample size of cases and examiners.

## CONCLUSION

APS depicted in CBCT is well-defined, shows low attenuation with curvilinear calcification and a sclerotic border, and has no effects on the surrounding structures. Familiarity with the APS CBCT radiographic features can improve OMFR confidence in the diagnosis.

OMFRs exposed to >20 CBCT cases of skull base per month were more confident in considering APS without further imaging than OMFRs who read <10 CBCT cases per month. These results have implications for CBCT continuous education for the OMFR, especially if their practice is limited to dentoalveolar imaging or minimal encounter with the skull base.

## Figures and Tables

**Table 1:** Descriptive radiographic features of arrested pneumatization of the sphenoid.

**Location**		**Periphery, Internal Structure, and Effects on Surrounding Structures**	
Unilateral	20 (69.0%)	Well defined	28 (96.6%)
Sphenoid body	27 (93.1%)	Corticated	28 (96.6%)
Greater wing of sphenoid	10 (34.5%)	Mixed attenuation with curvilinear calcification Low attenuation	28 (96.6%) 1 (3.4%)
Lesser wing of sphenoid	0 (0.0%)	Narrowing of vidian canal	1 (3.4%)
Pterygoid processes	19 (65.5%)	Expansion or erosion of cortical boundaries	0 (0.0%)
Clivus	1 (3.4%)	Displacement of foramina	0 (0.0%)
**Extension**		**Other Findings**	
Vidian canal	27 (93.1%)	Mucosal thickening	4 (13.8%)
Foramen rotundum	17 (58.6%)	-	-
Foramen ovale	1 (3.4%)	-	-
Carotid canal	1 (3.4%)	-	-
Foramen spinosum	0 (0.0%)	-	-

**Table 2:** Oral and maxillofacial radiologist (OMFR) inter-rater agreement and confidence in diagnosis.

**5 OMFR** **29 CBCT Cases**	**Inter-rater Agreement** **Kappa** **Standard Error (SE) [95% Confidence Interval]**				
	Expert*			**Not-Expert^**	
	OMFR1	OMFR2	OMFR3	OMFR4	OMFR5
OMFR1	-	0.73 SE 0.1 [0.5-1]	0.79 SE 0.1 [0.5-1]	0.05 SE 0.2 [-0.3- 0.^4^]	0.02 SE 0.2 [-0.3-3]
OMFR2	-	-	0.7 SE 0.2 [0.4-1.0]	-0.01 SE0.2 [-0.3-3]	0.10 SE 0.2 [-0.2-0.^5^]
OMFR3	-	-	-	0.02 SE 0.2 [-0.3-3]	0.14 SE 0.2 [-0.2-0.^4^]
OMFR4	-	-		-	0.78 SE 0.1 [0.55-1.00]
**Confident in Diagnosis** Yes (Y) No (N)	Y: 22 (75.9%) N: 7 (24.1%)	Y: 21 (72.4%) N: 8 (27.6%)	Y: 24 (82.8%) N: 5 (17.2%)	Y: 18 (62.1%) N: 11 (37.9%)	Y: 17 (58.6%) N: 12 (41.4%)
Absolute agreement of APS**	23 (79.3%)			15 (51.7%)	

**Note:** * > 20-30 Cone beam CT cases of skull base/ month

^ < 10 Cone beam CT cases of skull base/ month

**McNemar test p value= 0.05

APS: Arrested pneumatization of the sphenoid

**Table 3:** Logistic regression analysis of demographic and radiographic features affecting examiner’s decision making in arrested pneumatization of the sphenoid.

**Variable**	**Coefficient**	**Standard Error**	**Odds Ratio**	**95% Confidence Interval**	***P* value**
Age (child)	0.89	0.82	2.4	0.48 to 12.23	0.2
Extension to accessory sphenoid sites (No)	0.62	0.91	1.87	0.31 to 11.32	0.5
Extension to foramina (No)	-0.08	0.97	0.92	0.13 to 6.25	0.9
Location (bilateral)	-1.26	1.10	0.28	0.03 to 2.46	0.3
Constant	-0.60	0.87			0.5

**Note:** Full model -2 Log Likelihood= 35.5

Chi-squared=3.760, df=4, P = 0.4

Cox & Snell R^2^=0.12

Nagelkerke R^2^=0.16

Hosmer & Lemeshow test= 2.6893, df= 7, P= 0.9

## Data Availability

The data supporting this study's findings are available from the corresponding author, [N.A.], upon request.

## LIST OF ABBREVIATIONS

APS: Arrested Pneumatization Sphenoid
CBCT: Cone Beam Computed Tomography
OMFRs: Oral Maxillofacial Radiologists

## STANDARDS OF REPORTING

STROBE guidelines were followed.
